# Modeling Subjective Symptoms Related to Micro-Hydrargyrism in a Population of Moroccan Dentists

**DOI:** 10.4314/ejhs.v31i1.17

**Published:** 2021-01

**Authors:** Nourdine Attiya, Ayoub Filali, Rkia Fattahi, Soumia Moujane, Hamid Mazouz, Mohamed-Yassine Amarouch, Younes Filali-Zegzouti

**Affiliations:** 1 B.A.S.E Laboratory, Department of Biology, Faculty of Sciences and Techniques of Errachidia, Moulay Ismail University, Morocco; 2 Higher Institute of Nursing Profession and Techniques of Health, Kenitra, Morocco; 3 Faculty of Science, Ibn Tofail University, Kenitra, Morocco; 4 P.B.M.B Laboratory, Department of Biology, Faculty of Science, Moulay Ismail University, Meknes, Morocco; 5 R.N.E Laboratory, Multidisciplinary Faculty of Taza, Sidi Mohammed Ben Abdellah University, Fez, Morocco; 6 B.A.S.E Laboratory, Department of Biology, Faculty of Science, Moulay Ismail University, Meknes, Morocco

**Keywords:** Mercury exposure, Dental amalgam, Dentist, count data, Generalized linear models, Over-dispersion, Models selection

## Abstract

**Background:**

The ability of mercury to deposit throughout the body and alter a wide range of molecular and cellular pathways results in a polymorphic and complex clinical phenotype with over 250 possible symptoms. However, some of them are recurrently cited as evoking chronic mercury poisoning. In this light, dentists users of dental amalgams are chronically exposed to mercury so that in-depth epidemiological investigations and adapted statistical methods are required to highlight adverse effects of this exposure.

**Methods:**

In order to study the health impact of the occupational mercury exposure in a population of liberal dentists practicing in two Moroccan regions, a list of eighteen subjective symptoms commonly associated with micro-hydrargyrism was drawn up. Then, seven statisctical models adapted to count data were fitted. Finally, three methods were used to compare their relative performance in order to choose the most appropriate one.

**Results:**

The adopted logical path, from the response variable selection till models' comparison, led us to lean towards quasi-Poisson regression as the best way to predict the number of symptoms declared by dentists according to mercury exposure.

**Conclusions:**

Interpretation of the selected model allowed us to conclude that the reduction of dental amalgam use allows the reduction of subjective symptoms related to mercury exposure.

## Introduction

Mercury is the only metal found in a liquid state under ambient conditions. Owing to its very low vapor pressure, it readily vaporizes at room temperature. Under this state, it is easily absorbed through the lungs and distributed through the body via the bloodstream. Consequently, this metal is considered as a potent toxic biohazard at room temperatures (1). Moreover, due to its ability to deposit in many parts of the human body and to alter a wide array of molecular pathways, the resulting clinical picture of adverse effects of chronic mercury exposure is very complex and polymorphic. Indeed, chronic mercury poisoning is differently associated with over 250 symptoms and the clinical phenotype is never complete in each suspected case. Symptoms may appear, disappear, or persist depending on the duration and degree of exposure or its cessation (2). Moreover, mercury toxicity is known to be insidious and usually non-specific. Hence, the transition from acute to chronic poisoning may be silent (3). Nevertheless, literature review may help to draw up a list of non-specific symptoms that are commonly associated with chronic mercury poisoning (4).

In dentistry, elemental mercury is used in dental amalgams. Despite periodic safety concerns and the availability of alternative dental restorative materials, silver amalgam still remains one of the most cost- effective and durable material chosen by many dentists around the world (5). As handling metallic mercury is a well-known occupational hazard in many professions, dentists users of dental amalgam, are in risk of chronic mercury poisoning even though used doses are lower than in the other occupational fields (6). Consequently, correlations between symptoms and mercury exposure levels are more subtle to highlight and risk more difficult to assess. In this context, to correlate probable adverse effects related to mercury exposure within this population, indepth epidemiological investigations and adapted statistical methods to the particular nature of the generated clinical picture and the degree of exposure are required.

In this context, an exhaustive cross-sectional survey was carried out to evaluate mercury exposure among liberal dentists in two Moroccan regions (7). Descriptive analyses revealed two main sources of exposure. The most important one was associated with dental amalgam use (occupational exposure), and the second was related to the presence of dental amalgam fillings in the mouth (non-occupational exposure) (7).

In the present work, to investigate the health impact of this exposure within the studied population, a non-exhaustive list of eighteen symptoms that are commonly associated with chronic mercury poisoning was drawn up and integrated into the questionnaire distributed to participants (6–10). This list included: hyperpigmentation, eczematiform dematitis, heart palpitations or irregular pulse, shortness of breath, muscle or joint pain, salivation and metallic taste, painful chewing, vertigo, lack of movement coordination, fingers, lips or tongue tremor, anxiety and nervousness, depression, insomnia or sleep disturbance, chronic fatigue or lack of energy, memory deficits or concentration difficulties, and finally visual impairments with constriction of the visual field. The main objective of the present study is to model the relationship between the number of symptoms self-reported by the participants according to their mercury exposure. Thus, response variable (number of symptoms) is presented as a count and the purpose of the research is to find if a set of preselected explanatory variables integrated in a well-fitted statistical model could predict the number of symptoms declared by dentists.

## Material and Methods

**Study design and ethical considerations**: The current study uses a database obtained from a previous exhaustive cross-sectional survey conducted to evaluate the health impact of mercury exposure among a population of liberal dentists in two regions of central Morocco (7). This survey took place over a three-month period, from the beginning of February to the end of April 2016. A written call for participation, explaining objectives of the study, and a self-administered questionnaire were distributed to all liberal dentists in the studied regions. Moreover, verbal informed consents were obtained from all dentists who consented to participate in this study. Participation was voluntary, and confidentiality and anonymity were ensured by coding data collection sheets. From 271 dentists listed in the yellow pages, 192 were recruited in the survey (7).

**Initial selection of variables**: The main objective of this study is to understand whether, and how, the number of symptoms self-reported by dentists varies according to the occupational exposure to mercury. Thus, a non-exhaustive list of eighteen symptoms evoking chronic mercury poisoning was drawn up and integrated into the questionnaire. Moreover, an additional checkbox was dedicated to other probable unlisted symptoms that participants should specify. These additional symptoms were considered if they evoked mercury poisoning. A new variable (Tsym) was then created to code the total number of symptoms self-reported by each participant. Tsym is the random component of the model.

Six explanatory variables were initially selected:

- The number of amalgams weekly handled by each dentist (coded as Amalgamuse) to evaluate the current occupational exposure to mercury;
- The number of amalgam fillings in the participant mouth (coded as OwnAmalgam) to evaluate the nonoccupational exposure to mercury. The other exposure origins like fish consumption, vaccines or smoking were judged not relevant in our previous study (7);
- Four socio-demographic variables were also considered: gender, exercise area (urban or rural) coded as Area, seniority (number of years in the active practice) and age.

**Modeling count data; general remarks**: Conditions for Ordinary Least Square models (OLS) can rarely be assumed when outcomes are expressed as counts. Making awkwardly such a choice will lead to biased parameters as well as standard errors and confidence interval estimates of the wrong size (11). Moreover, negative values could be predicted by such model which is incompatible with counts. In this context, Poisson Regression (PR) is commonly used instead of OLS because it is a probability distribution designed for non-negative integers. Its canonical link is the log function resulting in a log-linear relationship between the mean and the linear predictors. However, PR assumes equality between the conditional mean and variance (dispersion parameter fixed at 1) (12). Due to this condition, PR exhibits overdispersion issue when the variance is greater than the mean. Over-dispersion induces small standard errors resulting in Wald tests of coefficients too large and overly liberal. It is therefore essential to deal with it before concluding about significance of correlations between dependent and explanatory variables (13). Quasi-Poisson (QP) and Negative Binomial (NB) distributions are commonly used in this case because they both have two parameters; the mean and a dispersion parameter greater than 1 (14). In addition to overdispersion, many real count datasets exhibit more zero observations than would be predicted by the Poisson model. In these situations, zero-inflated Poisson (ZIP) and zero-inflated negative binomial (ZINB) regressions are usually used. Poisson logit hurdle model (PLH) and negative binomial logit hurdle model (NBH) are two other alternatives that can also be used in such cases (11).

**Analysis strategy**: In order to build and choose the most appropriate model for our data, a five-step analysis strategy was defined:

- Graphical explorations of the data to inspect the distribution of the dependent variable and its relationships with each preselected explanatory variable;
- Fitting a first PR and defining the most appropriate systematic component;
- Basic PR model diagnostics based on residuals interpretation to identify particular observations and misspecification problems;
- As the basic PR was not equi-dispersed, six other models were then fitted to deal with over-dispersion and/or zero excess;
- Finally, three methods were then used to compare the relative adequacy of these different models in order to choose the most appropriate one.

**Fitted models and selection methods**: Seven models were fitted to the data: PR, QP, NB, ZIP, PLH, ZINB and NBH. In order to select the best of them, three methods were used to assess their relative performance:

- Comparison of the Akaike Information Criterion (AIC) and Bayesian Information Criterion (BIC) statistics;
- Comparison of the five-fold cross validation mean squared errors (CV.MSE);
- Graphical comparison using half-normal plots with a simulated envelope for residuals.

**Statistical software used for analyses**: The data analysis was carried out with the software R version 3.6.1 (15). The packages references are indicated at each step of the analysis.

## Results

**Exploratory data analyses**

**Figure 1:**
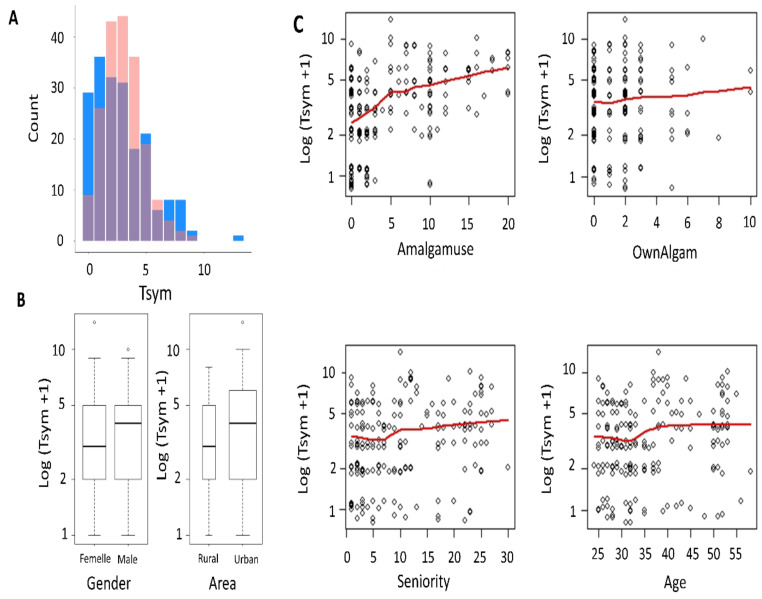
Data exploration: **A:** Barplots superposition showing differences between the frequency distribution of the response variable (in blue) and a randomly generated Poisson distribution (in red) using the same parameter (the mean) and number of observations **B:** Exploratory boxplots between log (response variable + 1) and the qualitative explanatory variables. As the log scale is used, 1 is added to avoid errors from log (0). **C:** Exploratory jittered scatterplots between log (response variable + 1) and quantitative independent variables. As the log scale is used, 1 is added to avoid errors from log (0). Lowess smoothed curves are created to judge linearity.

**Graphical exploration of the response variable**: A simple calculation of the mean (2.90) and the variance (5.71) of the response variable suggests that Poisson distribution would not be suitable. To confirm this, we compared our counts distribution with a randomly generated theoretical Poisson distribution with a parameter equal to the observed mean (Figure 1A). We notice that the theoretical Poisson distribution underestimates low and high counts and overestimates medium values. Moreover, the greatest value (13 symptoms) seems too far from the theoretical distribution and is, therefore, suspected to be an outlier.

**Bivariate graphical exploration**: Useful exploratory plots for count data include boxplots of the response when the predictor is a factor and scatterplots against quantitative predictors with a smooth nonparametric curve to detect possible nonlinearity. The obtained results are presented in Figure 1B and 1C). Lower number of symptoms is noticeable for rural and female dentists. Moreover, relationship between log(Tsym+1) and Amalgamuse seems largely linear except possibly at the very low end. In addition, the slope of the non-parametric line suggests a significant correlation if a loglinear regression model is fitted. Conversely, the nonparametric line with the other quantitative variables are almost horizontal suggesting weak correlations. Moreover, they appear almost identical for age and seniority which indicates probable collinearity.

**Variables selection and model specification for basic Poisson regression**: To select the most appropriate systematic component formula, a first Poisson regression using the main effects was fitted. First, the age was eliminated because of its high collinearity with seniority (Variance Inflation Factor (VIF) = 31.1 for both of them). Seniority was preferred to be kept in the model because it better reflects the time of occupational exposure rather than the simple age.

In order to determine at once the most relevant remaining terms and all pairwise interactions between them in PR, we have used the R package *glmulti* (16). AIC was chosen for selection, and relevant main effects with significant pairwise interactions were researched. The final formula contained two main effects (Amalgamuse and Area) and two interactions, between Amalgamuse on one hand and seniority and OwnAmalgam on the other. Thus, gender was dropped from the systematic component and seniority and OwnAmalgam kept only for their interactions with Amalgamuse.

This formula was run for PR, QP, NB and the count-part of ZIP, ZINB, PLH and NBH models. Additionally, a backward selection by the AIC criterion was performed for variable selection in the logit part of PLH and NBH. A logistic regression including only Amalgamuse with intercept was the most appropriate. Concerning ZIP and ZINB models, we used the R package *mpath* (17) for logit variables selection and decided to adopt the same formula as hurdle models.

**Figure 2:**
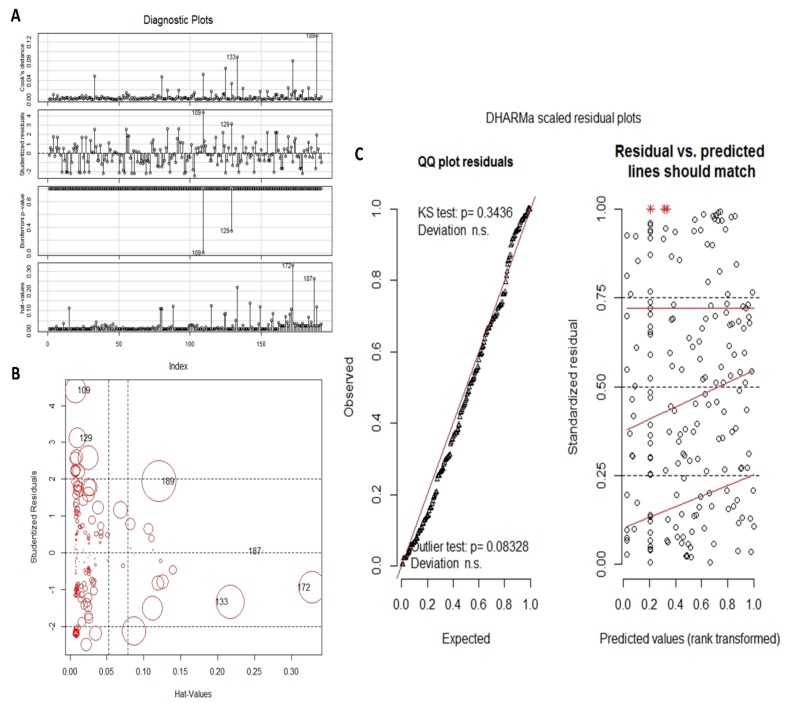
Basic Poisson regression diagnostics A: Diagnostic plots combining Cook's distance, studentized residuals, Bonferonni test p-values and hatvalues. Two farthest observations from average are numbered. B: Studentized residuals are plotted against hat-values, and the size of circle is proportional to Cook's distance. Particular observations are numbered. C: Scaled residuals exploration using the R package DHARMa. A qq-plot to detect overall deviations from the expected distribution (left hand) and a plot of the scaled residuals against the predicted value (right hand).

**Residuals and Poisson regression diagnostics**: The starting point for models selection was a PR fitted using the *glmulti* formula. It exhibited over-dispersion with a dispersion index of 1.63 and a highly significant deviance test which indicated an ill-fitting. Before investigating other models, residuals exploration of this first model was preliminary made to highlight observations with a large and undue influence on the analysis and to decide whether they should be kept or dropped. The R package *car* (18) was used to this aim. On the outputs (Figure 2A and 2B), we can note that the observation 109 (13 symptoms) has large studentized residual, moderate Cook's distance and very low hat-value. Thus, dropping this observation in modeling could help to reduce over-dispersion without having a noticeable effect on coefficients or standard errors while modeling. Indeed, without this observation, the improvement of the PR was significant and the dispersion index was lowered to 1.47 with coefficients and standard errors almost unchanged. Thus, we decided to drop definitively this observation in the further steps of analysis. The other particular observations were kept because it seemed to us that they were not more frequent than would be expected in a dataset of our size.

**Figure 3:**
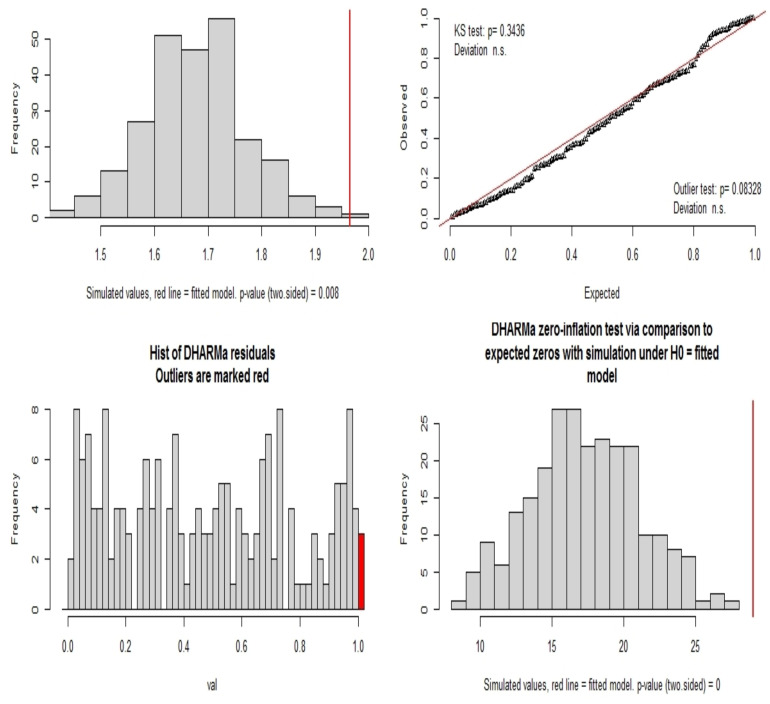
Goodness-of-fit tests using the R package DHARMa. Four tests were run: test of dispersion, test of uniformity, test for outliers and test of zero inflation

The R package *DHARMa* (19) was then used to continue the exploration because it seems more adapted for general linear models thanks to its scaled residuals. These latter are calculated using the “*simulateResiduals()”* function. The generated plots (Figure 2C) gave indications that the distribution was globally uniform but not equi-dispersed. Formal goodness-of-fit tests helped to confirm these observations (Figure 3). These tests showed that the model was uniform and without supplementary outliers but exhibited over-dispersion and zero inflation problems.

**Competing statistical models**: In order to ensure a structured approach in evaluating the competing models, a model building strategy was established with a PR as a starting point after defining the most appropriate systematic component and deleting one observation with evident outlier. Since PR exhibited overdispersion, we first investigated QP and NB models within the model selection process. PR was also unable to predict the whole number of zeros in the data. Thus, ZIP and PLH were considered to specially deal with this problem. ZINB and NBH were finally fitted to deal simultaneously with over-dispersion and zero excess.

**Figure 4:**
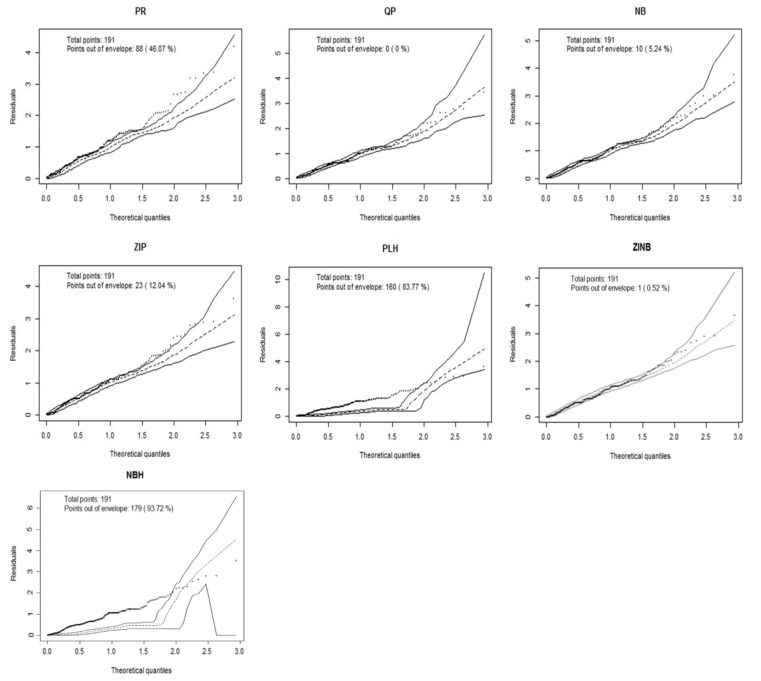
Half-normal plots with simulated envelopes for residuals from the seven competing models fitted in the study using hnp R package

**Model selection**: Model selection is a process of seeking the model in a set of candidate ones that gives the best balance between model fit and complexity. In the present study, three different methods were used to select the best model to predict the number of self-reported symptoms:

- AIC and BIC: The model with the smallest AIC was ZINB whereas with BIC, NB happened to be the best model. This difference is logical because BIC penalizes model complexity more heavily than does AIC.
- Five-fold Cross-Validation using *boot* package (20): Results revealed that QP has the smallest CV. MSE and therefore could be considered as the best model with this method.
- Half-normal plots with a simulated envelope for residuals using *hnp p*ackage (21): In Figure 4, we can conclude that QP is ideal because all residuals are lying within the simulated envelope.

These results allowed us to choose QP as the best way to model the number of symptoms declared by dentists in our population.

**Figure 5:**
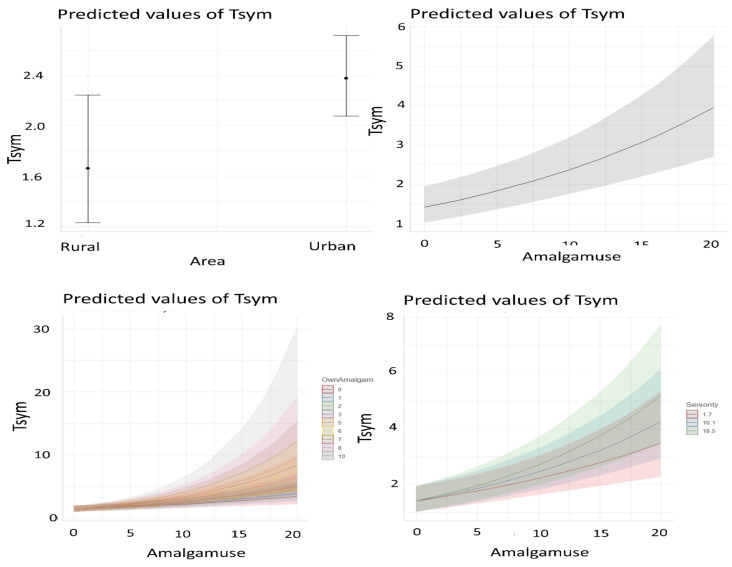
Prediction plots with confidence intervals of the number of symptoms self-reported by dentists according to each term in the systematic component

As the most appropriate model was selected, interpretation is made from its exponentiated coefficients which are all significant (Table 1). Since the model has interaction terms, the increase of symptoms number according to each term in the systematic component is much easier and more intuitive to observe on plots (Figure 5).

**Table 1:** Exponentiated coefficients (exp(β)), percentage change and *p*-values of the variables estimated in quasi Poisson regression model

	Quasi-Poisson		
Predictors	exp(β)*	%	p
(Intercept)	1.425	42.528	**0.024**
Area [Urban]	1.429	42.887	**0.002**
Amalgamuse	1.037	3.725	**0.002**
Amalgamuse : OwnAmalgam	1.006	0.630	**0.014**
Amalgamuse : Seniority	1.001	0.118	**0.002**

* Interpretation of estimates exp(β): a one unit change in the predictor variable is associated with a (exp(β) -1)*100) percentage increase of the expected number of symptoms, holding other variables constant, since β>0 for all predictors

## Discussion

The main purpose of this study was to identify how the frequent use of dental amalgam increases the number of subjective symptoms felt by dentists. For this aim, the presence or absence of these symptoms was treated as a count variable. Indeed, the clinical phenotype of chronic mercury poisoning is polymorphic: the absence of a symptom does not exclude the presence of another not systematically evoked by the first as in the classical situation where a homogeneous and formally suggestive clinical picture is present. Thus, in order to have a global overview on the clinical situation, symptoms were treated equally in the total count without investigating any systematic association between them as is done for formal diagnosis. This strategy allowed highlighting potential hidden risk that might be ignored by the classical approach.

Poisson regression model provides a basis for the analysis of count data. Many practitioners choose to use Poisson model when faced with statistical analysis involving count data even without ensuring that all assumptions of this model are met (11). The systematic way for choosing a model for fitting a particular data is that one should test whether the model's assumptions are met rather than just going the naïve way of fitting a model (22). Indeed, a well-fitted model is the culmination of several stages of exploration and comparison allowing the choice made to be assumed with a scientific-based conviction.

The deviance of a model always decreases with the inclusion of more predictors, but the presence of non-judicious predictors induces a larger variability in the estimation of the model which results in less precise prediction. Moreover, collinearity between regressors may hide significant coefficients or even change their signs (23). In this context, after pre-selecting explanatory variables, these two issues were the first to face after preliminary data exploration. The first step was therefore to select the most relevant variables and pairewise interactions to use in a basic Poisson model. As the fitting of this latter was bad, model's diagnostics were performed to recognize why and where the fit was poor. After deleting an observation with evident outlier and concluded that the model was over-dispersed and zero-inflated, six models were run to deal with these problems. In order to determine the most suitable of them, three different methods were used for their comparison.

The adopted logical path, from the response variable selection till the different models comparison, led us to choose QP model as the best way to predict the number of symptoms declared by dentists in correlation with mercury exposure. Its interpretation allowed us to conclude that the reduction or cessation of dental amalgam use helps to reduce significantly the number of subjective symptoms related to the occupational mercury exposure. Moreover, the duration and the presence of another source of exposure contributes to increasing the number of symptoms expressed by dentists.

The present study, despite methodological precautions taken while modeling and the interest of the obtained results, suffers from two main problems. Indeed, as our dependent variable was based only on the self-reported symptoms from a pre-established list, a classification bias might be generated by the suggestibility of some participants or, oppositely, by their misinterpretation of long-lasting symptoms. In the systematic component of the fitted model, exposure was estimated only by the number of amalgams weekly handled which reflects globally, without great precision, the current exposure. In this context and in the light of the encouraging obtained results, another study with an objective determination of symptoms (e.g. neuro-behavioral tests or adapted and validated questionnaires) and a more precise evaluation of the current and cumulative (body burden) exposure is highly desirable.
